# Synaptome.db: a bioconductor package for synaptic proteomics data

**DOI:** 10.1093/bioadv/vbac086

**Published:** 2022-11-12

**Authors:** Oksana Sorokina, Anatoly Sorokin, J Douglas Armstrong

**Affiliations:** School of informatics, University of Edinburgh, Edinburgh EH8 9AB, UK; Department of Biochemistry and Systems Biology, Institute of Systems, Molecular and Integrative Biology, Faculty of Health and Life Sciences, University of Liverpool, Liverpool, UK; Biological Systems Unit, Okinawa Institute of Science and Technology, Okinawa 1919-1, Japan; School of informatics, University of Edinburgh, Edinburgh EH8 9AB, UK; Computational Biomedicine Institute (IAS-5/INM-9), Forschungszentrum Jülich, Jülich 52428, Germany

## Abstract

**Summary:**

The neuronal synapse is underpinned by a large and diverse proteome but the molecular evidence is spread across many primary datasets. These data were recently curated into a single dataset describing a landscape of ∼8000 proteins found in studies of mammalian synapses. Here, we describe programmatic access to the dataset via the R/Bioconductor package Synaptome.db, which enables convenient and in-depth data analysis from within the Bioconductor environment. Synaptome.db allows users to obtain the respective gene information, e.g. subcellular localization, brain region, gene ontology, disease association and construct custom protein–protein interaction network models for gene sets and entire subcellular compartments.

**Availability and implementation:**

The package Synaptome.db is part of Bioconductor since release 3.14, https://bioconductor.org/packages/release/data/annotation/html/synaptome.db.html, it is open source and available under the Artistic license 2.0. The development version is maintained on GitHub (https://github.com/lptolik/synaptome.db). Full documentation including examples is provided in the form of vignettes on the package webpage.

**Supplementary information:**

Supplementary data are available at *Bioinformatics Advances* online.

## 1 Introduction

The proteomes of the presynaptic and postsynaptic compartments mediate information processing in the brain via complex and highly dynamic molecular networks. Sorokina *et al.* (2021) systematically curated 58 proteomic studies from 2000 to 2020, to produce a comprehensive dataset describing >8000 proteins expressed at the mammalian synapse (Sorokina *et al.*, 2021). The set includes 29 post-synaptic proteome (PSP) studies (2000–2019) contributing to a total of 5560 mouse, human and rat unique gene identifiers; 18 presynaptic studies (2004–2020) resulting in 2772 unique gene IDs and 11 studies for whole synaptosomes reporting 7198 unique gene IDs.

**Fig. 1.**
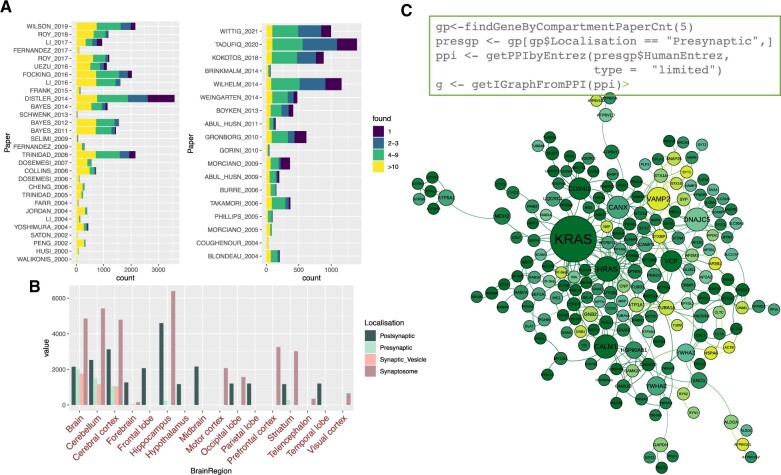
Summary statistics for data combined in Synaptome.data and an example of the functionality of Synaptome.db. (**A**) Studies used for aggregation of pre- (right) and post-synaptic (left) compartments with numbers of identified proteins; node shade represents distribution of discovery rate for proteins found in each study, where yellow corresponds to the highly confident proteins, identified in at *n* > 10 studies and dark blue corresponds to the proteins found in just a single study. (**B**) Distribution of numbers of proteins by brain region; color corresponds to distribution of subcellular localization for the proteins in each of the region **C**) Code example for extraction of a presynaptic PPI network and the resulting graph visualized with Gephi (https://gephi.org/), node shading is conserved across the elements A and C of the figure, node size corresponds to node degree

Each synaptic component was annotated with relevant metadata based on the respective study (author, year, method, subcellular compartment and brain region) and associated with function and disease information according to Gene Ontology and Human Disease Ontology. Figure 1A shows studies aggregating pre- (right panel) and postsynaptic (left panel) compartments with numbers of identified proteins, while Figure 1B shows the brain regions, annotated from the studies with respective numbers of proteins. It could be seen that coverage highly varies between regions, as the most of collected studies were performed on the whole brain, hippocampus, cerebellum and cerebral cortex.

Furthermore, the protein–protein interactions (PPI) were obtained for the pre- and post-synaptic proteomes based on combined human, mouse and rat data from BioGRID (Oughtred *et al.*, 2019), Intact (Kerrien *et al.*, 2012) and DIP (Salwinski *et al.*, 2004). Interaction sources were filtered for methods that produce data on direct physical interactions with the highest confidence. The interaction data from each database was extracted in the PSI-MITAB format.

To merge the datasets, we standardized the IDs used, by mapping each onto Entrez gene IDs. To extract only direct interactions, the ‘interaction type’ column was then filtered for the PSI-MI terms ‘association’ (MI: 0914), ‘physical association’ (MI: 0915) and ‘direct interaction’ (MI: 0407) and their 63 child-terms. Some of the source data used an obsolete interaction type MI: 0218, ‘physical interaction’ which could still be used, since it was updated to the association and physical association, which we both include. PPIs based on the interaction types: ‘genetic interaction’ (MI: 0208) [including ‘suppression’ (MI: 0796) and ‘synthetic’ (MI: 0794)], ‘colocalization’ (MI: 0403), ‘genetic interference’ (MI: 0254) and ‘additive genetic interaction defined by inequality’ (obsolete term, MI: 0799) were excluded from the final set as these methods are designed to include both direct and indirect interactions.

To maximize confidence in direct physical interactions, we also excluded predicted interactions and interactions obtained by Co-IP experiments (spoke models), filtering out the PSI-MI terms like ‘Pull-down’, ‘Affinity technology’, etc.

This resulted in two large-scale PPI networks (4817 nodes and 27 788 edges for PSP and 2221 nodes and 8678 edges for presynaptic proteome).

Combined these provide a unified and configurable resource for constructing customized networks for the synaptic proteome. The resulting network model is available in a SQLite implementation from Edinburgh DataShare https://doi.org/10.7488/ds/3771 and EBRAINS.

Although highly extendible, the SQLite implementation requires specific database-related expertise restricting its use to specialist bioinformatics researchers; while gene information and network models stored in the database provide the much-in-demand resource for the broader community of the molecular neuroscientists. To make the database more widely accessible we developed the Bioconductor package synaptome.db, which enables direct access to the data (embedded into the satellite package synaptome.data) from within the R environment. We developed both packages as components of Bioconductor project (Salwinski *et al.*, 2004), which is designed to facilitate rigorous and reproducible analysis of biological data by building customized pipelines and workflows (Huber *et al.*, 2015). The incorporation into Bioconductor allows users to combine the synaptic PPI networks and protein annotation with external genomics (org.Hs.eg.db, org. Mm.eg.db and org. Rn.eg.db packages (https://bioconductor.org/packages/release/data/annotation/html/org.Hs.eg.db.html; https://bioconductor.org/packages/release/data/annotation/html/org.Mm.eg.db.html; https://bioconductor.org/packages/release/data/annotation/html/org.Rn.eg.db.html)), transcriptomics (via various ChipDB packages (https://bioconductor.org/packages/release/BiocViews.html#___ChipDb)), mutations and polymorphism analysis (via PolyPhen.Hsapiens.dbSNP131 (https://bioconductor.org/packages/release/data/annotation/html/PolyPhen.Hsapiens.dbSNP131.html)) to name just a few examples. Results of analysis could be presented in a domain-specific manner by, for example, ggbio (Yin *et al.*, 2012) or KaryoploteR packages (Gel and Serra, 2017) (see the example below). Synaptome.db can be also used to provide annotation for experimental datasets, or as a source for hypothesis generation and experimental design.

## 2 Implementation

To comply with the requirements of Bioconductor, the database itself was wrapped into an AnnotationHub (Morgan and Shepherd, 2021) package, synaptome.data that fetches the most recent version of the database from Edinburgh DataShare site and caches it for further use. The synaptome.db package provides a simple API for extracting the data from the database without the understanding of the underlying database structure or using other database-related skills. Users with SQL experience can still also query the database directly via synaptome.data package using the schema described in Sorokina *et al.* (2021).

### 2.1 Synaptome.db functionality

The functions implemented in the current release were designed to support the most frequent user queries: When?, and by whom?, was my favorite gene (or list of genes) identified? Was my gene/list found pre- or post-synaptically? and how often? Was it found in a specific brain region? and which diseases it is associated with?

Functions findGenesByEntrez and findGenesByName return the following identifiers for genes specified by Entrez ID or gene name, respectively: GeneID (internal database ID), MGI ID, Human Entrez ID, Mouse Entrez ID, Rat Entrez ID, Human gene name, Mouse gene name and Rat gene name. Here, Internal GeneID corresponds to our unique database ID, which helps to resolve ambiguity across the external IDS, for example where a mouse Entrez gene IDs matches the same Human one, etc. Internal GeneIDs can then be used to extract subcellular compartment (getAllGenes4 Compartment) or brain region (getAllGenes4BrainRegion) protein composition, and for extracting PPIs for selected molecules (getPPIbyIDs), as shown in Figure 1. It is also possible to get Human disease information (HDO provided) for any subset of Human Entrez IDs (getGeneDiseaseByEntres), internal Gene IDs and Human gene names. As it is based on manually curated data, synaptome.db provides a literature provenance trail (getGeneInfoByIDs) for each of its data points, including details such as Localization (one of the following: presynaptic, post-synaptic and synaptosome), PaperPMID (PMID for the publications where the genes were reported), Paper (papers where specific genes were reported in a format FIRSTAUTHOR_YEAR), Year, SpeciesTaxID (species on which the original experiment was performed on) and BrainRegion (Brain region where the specific genes were identified, according to the paper).

Where a user’s wants to check whether a query set of proteins have previously been identified as synaptic, we enabled a quick check by command getGenes4Compartment and getGenes4BrainRegion, where one needs to provide Compartment Id and Specie TaxID or/and BrainRegion ID, along with the list of internal Gene Ids for the proteins obtained from experiment.

Given that the diversity across synaptic proteomics datasets (e.g. low overlap between some synaptosome datasets) could easily be due to differences in biochemical enrichment protocols and mass-spec setups, it is likely that only a subset of proteins in each dataset described here are truly synaptic. Figure 1A demonstrates the distribution of proteins with different discovery rates over pre- and post-synaptic studies. It could be seen that most stable (yellow) population makes more or less regular proportion, while the number of proteins discovered only in single studies (dark blue) varies between the datasets. To tackle this issue, we enabled a few functions that use ‘count’ (discovery rate, or number of protein identification in different studies) to enable custom filters for the proteins that were identified more frequently than others, thus, may correspond to more probable synaptic residents. One of them, findGeneByPaperCnt, selects the proteins from the total list off ∼8000, which were found more than defined ‘count’ of studies, e.g. one can select the genes that were identified in more than five studies in all compartments. Another, findGeneByCompartmentPaperCnt, allows similar filtering for specific compartment.

The use of this command in illustrated in Figure 1C, where we selected the most confident protein set (e.g. ‘count’ = 5, proteins identified in at least five presynaptic studies). In addition, the command findGeneByPapers enables the extraction of protein lists from specific studies, which can be listed with the command getPapers.

Finally, the package supports the extraction of PPIs for the gene list or entire compartment/brain region and their export in a form of a network graph or a table (example code and network presented in Figure 1C). Custom PPIs based on bespoke subsets of molecules can be extracted in two general ways: ‘induced’ and ‘limited’. In the first case, the command will return all possible interactors for the genes within the whole interactome. In the second case, it will return only interactions between the genes of interest. PPIs could be obtained by submitting list of EntrezIDs or gene names, or Internal IDs—in all cases the interactions will be returned as a list of interacting pairs of Internal GeneIDs.

To summarize, the package allows users to do the following:

- Finding a variety of Gene ID information for specific gene/lists(s)
- Finding molecular composition for specific compartments or brain regions
- Finding the most confident set of proteins for the total synaptosome or specific compartments
- Extracting the protein lists from specific papers
- Finding disease associations for selected genes
- Comparing user-defined protein lists against specific compartments and/or brain regions
- Finding PPIs for selected genes/compartments/brain regions.
- Constructing custom PPI graphs and network models

(See Supplementary materials for package vignette and manual with detailed functionality.)

## 3 Example

**Fig. 2.**
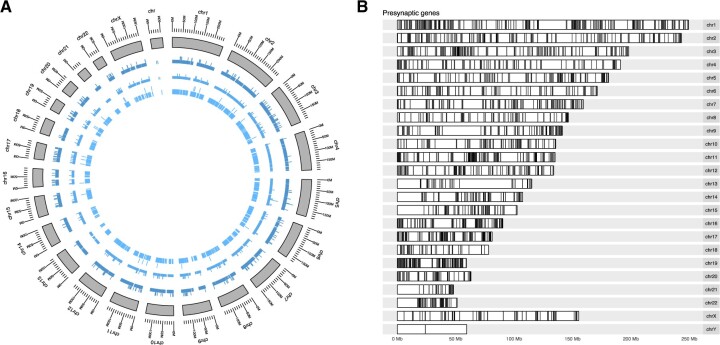
Distribution of synaptic genes over the human chromosomes. (**A**) Circus diagram showing the distribution of pre-, post- and synaptosome genes on each chromosome. (**B**) The localization of presynaptic genes on the Human chromosomes

The following brief example demonstrates how the SynaptomeDB can be used in combination with other Bioconductor packages (Fig. 2).

We extracted a complete list of human gene IDs for each of the presynaptic compartment, the postsynaptic compartment and the entire synaptosome. For each of these gene sets, we mapped genes onto the Human kariotype to get a distribution map of the respective gene positions across all human chromosomes using the ggbio package (Yin *et al.*, 2012).

We could then select genes that are annotated to any specific disorder, e.g. Alzheimer’s disease (AD). Supplementary Figure S1 shows the distribution of AD-related synaptic genes across human chromosomes. The color code corresponds to each gene’s subcellular localization. R code for the example is available from Supplementary materials.

## 4 Conclusions

We developed the Bioconductor packages synaptome.data and synaptome.db to provide a simple and intuitive access to the data in SynaptomeDB. These packages can easily be incorporated into custom bioinformatics data pipelines along with other annotations, experimental data and statistical methods exploiting the features of Bioconductor and R for further analysis. We aim to update the package twice a year to incorporate newly available datasets and are open to suggestions.

## Supplementary Material

vbac086_supplementary_data

## Data Availability

No new data were generated or analysed in support of this research.
