# Supplementary material for: Synaptome.db: a bioconductor package for synaptic proteomics data
Source: Bioinform Adv. 2022 Nov 12;2(1):vbac086. doi: 10.1093/bioadv/vbac086 (PMC9710567; doi:10.1093/bioadv/vbac086)
Supplement: vbac086_supplementary_data [file vbac086_supplementary_data.zip › suppl_data/supplementary.pdf]

SynaptomeDB: database for Synaptic proteome

Manual for querying SynaptomeDB

Oksana Sorokina, Anatoly Sorokin, J. Douglas Armstrong

21.10.2022

Introduction

64 published synaptic proteomic datasets (2000-2022) that describe over 8,000 proteins were integrated and combined with direct protein-protein interactions and functional metadata to build a network resource.

Current set includes 29 post synaptic proteome (PSP) studies (2000 to 2019) contributing a total of 5,560 mouse and human unique gene identifiers; 19 presynaptic studies (2004 to 2022) describe 2,853 unique human and mouse gene IDs, and 11 studies that span the whole synaptosome and report 7,528 unique genes. We considered also a Synaptic Vesicle compartment as a separate one, with 11 studies (a subset of Presynaptic list). In addition, we have annotated genome variations linked to Autistic Spectral Disorder (ASD) and Epilepsy (Epi).

To reconstruct protein-protein interaction (PPI) networks for the pre- and post-synaptic proteomes we used human PPI data filtered for the highest confidence direct and physical interactions from BioGRID, Intact and DIP. The resulting postsynaptic proteome (PSP) network contains 4,817 nodes and 27,788 edges in the Largest Connected Component (LCC). The presynaptic network is significantly smaller and comprises 2,221 nodes and 8,678 edges in the LCC.

The database includes: proteomic and interactomic data with supporting information on compartment, specie and brain region, GO function information for three species: mouse, rat and human, disease annotation for human (based on Human Disease Ontology (HDO), and mutations for ASD and Epi.

The original files are maintained at Ednburgh Datashare https://doi.org/10.7488/ds/3017. Updated database file could be found here: https://doi.org/10.7488/ds/3771

The dataset was described in the @Sorokina:2021hl.

Overview of capabilities

```
suppressMessages(library(synaptome.db))
suppressMessages(library(dplyr))
library(ggplot2)
library(pander)
```

1.Get information for a specific gene or gene set.

The dataset can be used to answer frequent questions such as “What is known about my favourite gene? Is it pre- or postsynaptic? Which brain region was it identified in?”, “Which publication it was reported in?” Information could be obtained by submitting gene EntrezID or Gene name

```
t <- getGeneInfoByEntrez(1742)
pander(head(t))
```

Таблица 1: Table continues below

GeneID	Localisation	MGI	HumanEntrez	MouseEntrez	HumanName
1	Postsynaptic	MGI:1277959	1742	13385	DLG4
1	Postsynaptic	MGI:1277959	1742	13385	DLG4
1	Postsynaptic	MGI:1277959	1742	13385	DLG4
1	Postsynaptic	MGI:1277959	1742	13385	DLG4
1	Postsynaptic	MGI:1277959	1742	13385	DLG4
1	Postsynaptic	MGI:1277959	1742	13385	DLG4

MouseName	PaperPMID	Paper	Year	SpeciesTaxID	BrainRegion
Dlg4	10818142	WALIKONIS_2000	2000	10116	Forebrain
Dlg4	10862698	HUSI_2000	2000	10090	Forebrain
Dlg4	11895482	SATON_2002	2002	10090	Forebrain
Dlg4	14532281	LI_2004	2004	10116	Forebrain
Dlg4	14720225	YOSHIMURA_2004	2004	10116	Forebrain
Dlg4	15020595	PENG_2002	2004	10116	Forebrain

```
t <- getGeneInfoByName("CASK")
pander(head(t))
```

Таблица 3: Table continues below

GeneID	Localisation	MGI	HumanEntrez	MouseEntrez	HumanName
409	Postsynaptic	MGI:1309489	8573	12361	CASK
409	Postsynaptic	MGI:1309489	8573	12361	CASK
409	Postsynaptic	MGI:1309489	8573	12361	CASK
409	Postsynaptic	MGI:1309489	8573	12361	CASK
409	Postsynaptic	MGI:1309489	8573	12361	CASK
409	Postsynaptic	MGI:1309489	8573	12361	CASK

MouseName	PaperPMID	Paper	Year	SpeciesTaxID	BrainRegion
Cask	15169875	JORDAN_2004	2004	10090	Brain
Cask	16635246	COLLINS_2006	2006	10090	Forebrain
Cask	17623647	DOSEMESI_2007	2007	10116	Cerebral cortex
Cask	18056256	TRINIDAD_2008	2008	10090	Midbrain
Cask	18056256	TRINIDAD_2008	2008	10090	Cerebellum
Cask	18056256	TRINIDAD_2008	2008	10090	Hippocampus

```
t <- getGeneInfoByName(c("CASK", "DLG2"))
pander(head(t))
```

Таблица 5: Table continues below

GeneID	Localisation	MGI	HumanEntrez	MouseEntrez	HumanName
6	Postsynaptic	MGI:1344351	1740	23859	DLG2

GeneID	Localisation	MGI	HumanEntrez	MouseEntrez	HumanName
6	Postsynaptic	MGI:1344351	1740	23859	DLG2
6	Postsynaptic	MGI:1344351	1740	23859	DLG2
6	Postsynaptic	MGI:1344351	1740	23859	DLG2
6	Postsynaptic	MGI:1344351	1740	23859	DLG2
6	Postsynaptic	MGI:1344351	1740	23859	DLG2

MouseName	PaperPMID	Paper	Year	SpeciesTaxID	BrainRegion
Dlg2	10862698	HUSI_2000	2000	10090	Forebrain
Dlg2	11895482	SATON_2002	2002	10090	Forebrain
Dlg2	14532281	LI_2004	2004	10116	Forebrain
Dlg2	14720225	YOSHIMURA_2004	2004	10116	Forebrain
Dlg2	15169875	JORDAN_2004	2004	10090	Brain
Dlg2	15748150	TRINIDAD_2005	2005	10090	Brain

2.Get internal GeneIDs for the list of genes.

Obtaining Internal database GeneIDs is a useful intermediate step for more complex queries including those for building protein-protein interaction (PPI) networks for compartments and brain regions. Internal GeneID is specie-neutral and unique, which allows exact identification of the object of interest in case of redundancy (e.g. one Human genes matches on a few mouse ones, etc.)

```
t <- findGenesByEntrez(c(1742, 1741, 1739, 1740))
pander(head(t))
```

Таблица 7: Table continues below

GeneID	MGI	HumanEntrez	MouseEntrez	RatEntrez	HumanName
1	MGI:1277959	1742	13385	29495	DLG4
6	MGI:1344351	1740	23859	64053	DLG2
15	MGI:1888986	1741	53310	58948	DLG3
46	MGI:107231	1739	13383	25252	DLG1

MouseName	RatName
Dlg4	Dlg4
Dlg2	Dlg2
Dlg3	Dlg3
Dlg1	Dlg1

```
t <- findGenesByName(c("SRC", "SRCIN1", "FYN"))
pander(head(t))
```

Таблица 9: Table continues below

GeneID	MGI	HumanEntrez	MouseEntrez	RatEntrez	HumanName
48	MGI:1933179	80725	56013	56029	SRCIN1
585	MGI:98397	6714	20779	83805	SRC

GeneID	MGI	HumanEntrez	MouseEntrez	RatEntrez	HumanName
710	MGI:95602	2534	14360	25150	FYN

MouseName	RatName
Srcin1<br>Src<br>Fyn	Srcin1<br>Src<br>Fyn

3.Get disease information for the gene set

Synaptic genes are annotated with disease information from Human Disease Ontology, where available. To get disease information one can submit the list of Human Entrez Is or Human genes names, it could be also the list of Internal GeneIDs if using getGeneDiseaseByIDs function

```
t <- getGeneDiseaseByName (c("CASK", "DLG2", "DLG1"))
pander(head(t))
```

HumanEntrez	HumanName	HDOID	Description
1740	DLG2	DOID:936	brain_disease
8573	CASK	DOID:936	brain_disease
1739	DLG1	DOID:331	central_nervous_system_disease
1740	DLG2	DOID:331	central_nervous_system_disease
8573	CASK	DOID:331	central_nervous_system_disease
1739	DLG1	DOID:863	nervous_system_disease

```
t <- getGeneDiseaseByEntres (c(8573, 1742, 1739))
pander(head(t))
```

HumanEntrez	HumanName	HDOID	Description
8573	CASK	DOID:936	brain_disease
1739	DLG1	DOID:331	central_nervous_system_disease
1742	DLG4	DOID:331	central_nervous_system_disease
8573	CASK	DOID:331	central_nervous_system_disease
1739	DLG1	DOID:863	nervous_system_disease
1742	DLG4	DOID:863	nervous_system_disease

5.Get information about the studies, combined into dataset

One can obtain the overview of synaptic proteome papers combined into the database, which includes paper PMID, specie Tax ID, year of publication, subcellular localisation, brain region and number of proteins identified in the paper. This information may help to choose the specific study(ies) for further work.

```
p <- getPapers()
pander(head(p))
```

Таблица 13: Table continues below

PaperPMID	SpeciesTaxID	Year	Name	Localisation	BrainRegion
10818142	10116	2000	WALIKONIS_2000	Postsynaptic	Forebrain
10862698	10090	2000	HUSI_2000	Postsynaptic	Forebrain
11895482	10090	2002	SATON_2002	Postsynaptic	Forebrain
14532281	10116	2004	LI_2004	Postsynaptic	Forebrain
14720225	10116	2004	YOSHIMURA_2004	Postsynaptic	Forebrain
15007177	10116	2004	BLONDEAU_2004	Presynaptic	Brain

Method	Ngenes
Shotgun	29
Shotgun	77
Shotgun	45
Shotgun	138
Shotgun	436
Shotgun	209

6.Get the table of frequently identified proteins

It is also possible to obtain the list of proteins found in more than one study, for the whole synaptic proteome. For that, the user needs to provide a "count" value as a desired minimal number of identifications (e.g. 2 or more). The command returns the table with gene identifiers and "Npmid" column, which contains the number of studies where this gene was identified.

```
gp <- findGeneByPaperCnt(cnt = 2)#find all proteins in synaptic proteome identified 2 times or more
#> Warning in result_fetch(res@ptr, n = n): Column `RatEntrez`: mixed type, first
#> seen values of type integer, coercing other values of type string
pander(head(gp))
```

Таблица 15: Table continues below

GeneID	MGI	HumanEntrez	MouseEntrez	RatEntrez	HumanName
1	MGI:1277959	1742	13385	29495	DLG4
2	MGI:88256	815	12322	25400	CAMK2A
3	MGI:96568	9118	226180	24503	INA
4	MGI:98388	6711	20742	305614	SPTBN1
5	MGI:88257	816	12323	24245	CAMK2B
6	MGI:1344351	1740	23859	64053	DLG2

MouseName	RatName	Npmid
Dlg4	Dlg4	48
Camk2a	Camk2a	54
Ina	Ina	50
Sptbn1	Sptbn1	45
Camk2b	Camk2b	46
Dlg2	Dlg2	43

7.Get the table of proteins identified in specific studies

Following section 5, when the information for all considered proteomic studies was obtained, user can select specific study(ies) by PMID and get the proteins identified in those studies. By providing "count" value user can extract either all proteins from specified studies (count = 1), or just frequently found ones (count >=2). As above, the command returns the table with gene identifiers with "Npmid" column, which contains the number of studies where this protein was identified

```
spg <- findGeneByPapers(p$PaperPMID[1:5], cnt = 1)
pander(head(spg))
```

Таблица 17: Table continues below

GeneID	MGI	HumanEntrez	MouseEntrez	RatEntrez	HumanName
1	MGI:1277959	1742	13385	29495	DLG4
2	MGI:88256	815	12322	25400	CAMK2A
3	MGI:96568	9118	226180	24503	INA
4	MGI:98388	6711	20742	305614	SPTBN1
5	MGI:88257	816	12323	24245	CAMK2B
6	MGI:1344351	1740	23859	64053	DLG2

MouseName	RatName	Npmid
Dlg4	Dlg4	5
Camk2a	Camk2a	5
Ina	Ina	5
Sptbn1	Sptbn1	4
Camk2b	Camk2b	4
Dlg2	Dlg2	4

8.Get the table of proteins frequently identified in specific compartment

Most of the times, user is interested in the specific compartment rather then in total synaptic proteome. To help identify the genes most probably residenting in the specific compartment and exclude possible contaminants, findGeneByCompartmentPaperCnt function provides the table of proteins found "cnt" or more times in different compartment-paper pairs.

```
gcp <- findGeneByCompartmentPaperCnt(cnt = 2)
#> Warning in result_fetch(res@ptr, n = n): Column `RatEntrez`: mixed type, first
#> seen values of type integer, coercing other values of type string
pander(head(gcp))
```

Таблица 19: Table continues below

GeneID	MGI	HumanEntrez	MouseEntrez	RatEntrez	HumanName
1	MGI:1277959	1742	13385	29495	DLG4
1	MGI:1277959	1742	13385	29495	DLG4
1	MGI:1277959	1742	13385	29495	DLG4
1	MGI:1277959	1742	13385	29495	DLG4
2	MGI:88256	815	12322	25400	CAMK2A
2	MGI:88256	815	12322	25400	CAMK2A

MouseName	RatName	Localisation	Npmid
Dlg4	Dlg4	Postsynaptic	29
Dlg4	Dlg4	Presynaptic	4
Dlg4	Dlg4	Synaptosome	16
Dlg4	Dlg4	Synaptic_Vesicle	3
Camk2a	Camk2a	Postsynaptic	28
Camk2a	Camk2a	Presynaptic	13

Now user can select the specific compartment and proceed working with obtained list of frequently found proteins

```
presgp <- gcp[gcp$Localisation == "Presynaptic",]
dim(presgp)
#> [1] 1542   10
pander(head(presgp))
```

Таблица 21: Table continues below

GeneID	MGI	HumanEntrez	MouseEntrez	RatEntrez	HumanName
1	MGI:1277959	1742	13385	29495	DLG4
2	MGI:88256	815	12322	25400	CAMK2A
3	MGI:96568	9118	226180	24503	INA
4	MGI:98388	6711	20742	305614	SPTBN1
5	MGI:88257	816	12323	24245	CAMK2B
6	MGI:1344351	1740	23859	64053	DLG2

MouseName	RatName	Localisation	Npmid
Dlg4	Dlg4	Presynaptic	4
Camk2a	Camk2a	Presynaptic	13
Ina	Ina	Presynaptic	12
Sptbn1	Sptbn1	Presynaptic	8
Camk2b	Camk2b	Presynaptic	8
Dlg2	Dlg2	Presynaptic	3

9.Get PPI interactions for my list of genes

Custom Protein-protein interactions based on bespoke subsets of molecules could be extracted in two general ways: “induced” and “limited”. In the first case, the command will return all possible interactors for the genes within the whole interactome. In the second case it will return only interactions between the genes of interest. PPIs could be obtained by submitting list of EntrezIDs or gene names, or Internal IDs - in all cases the interactions will be returned as a list of interacting pairs of Intenal GeneIDs.

```
t <- getPPIbyName(
    c("CASK", "DLG4", "GRIN2A", "GRIN2B","GRIN1"),
    type = "limited")
pander(head(t))
```

A	B
38	1
7	1

A	B
1	7
1	38
1	9
9	1

```
t <- getPPIbyEntrez(c(1739, 1740, 1742, 1741), type='induced')
pander(head(t))
```

A	B
1	2871
6	2871
15	2871
1	617
1	30
156	1

```
#obtain PPIs for the list of frequently found genes in presynaptc compartment
t <- getPPIbyEntrez(presgp$HumanEntrez, type='induced')
pander(head(t))
```

A	B
365	148
1048	148
52	365
52	1048
321	1048
321	365

10.Get the molecular structure of synaptic compartment

Three main synaptic compartments considered in the database are “presynaptic”, “postsynaptic” and “synaptosome”. Genes are classified to compartments based on respective publications, so that each gene can belong to one or two, or even three compartments. The full list of genes for specific compartment could be obtained with command getAllGenes4Compartment, which returns the table with main gene identifiers, like internal GeneIDs, MGI ID, Human Entrez ID, Human Gene Name, Mouse Entrez ID, Mouse Gene Name, Rat Entrez ID, Rat Gene Name.

If you need to check which genes of your list belong to specific compartment, you can use getGenes4Compartment command, which will select from your list only genes associated with specific compartment. To obtain the PPI network for compartment one has to submit the list of Internal GeneIDs obtained with previous commands.

```
#getting the list of compartment
comp <- getCompartments()
pander(comp)
```

ID	Name	Description
1	Postsynaptic	Postsynaptic

ID	Name	Description
2	Presynaptic	Presynaptic
3	Synaptosome	Synaptosome
4	Synaptic_Vesicle	Synaptic_Vesicle

```
#getting all genes for postsynaptic compartment
gns <- getAllGenes4Compartment(compartmentID = 1)
pander(head(gns))
```

Таблица 27: Table continues below

GeneID	MGI	HumanEntrez	MouseEntrez	RatEntrez	HumanName
1	MGI:1277959	1742	13385	29495	DLG4
2	MGI:88256	815	12322	25400	CAMK2A
3	MGI:96568	9118	226180	24503	INA
4	MGI:98388	6711	20742	305614	SPTBN1
5	MGI:88257	816	12323	24245	CAMK2B
6	MGI:1344351	1740	23859	64053	DLG2

MouseName	RatName
Dlg4	Dlg4
Camk2a	Camk2a
Ina	Ina
Sptbn1	Sptbn1
Camk2b	Camk2b
Dlg2	Dlg2

```
#getting full PPI network for postsynaptic compartment
ppi <- getPPIbyIDs4Compartment(gns$GeneID,compartmentID =1, type = "induced")
pander(head(ppi))
```

A	B
365	148
1048	148
52	365
52	1048
321	1048
321	365

11.Get the molecular structure of the brain region.

Three are 12 brain regions considered in the database based on respective publications, so that each gene can belong to the single or to the several brain regions. Brain regions differ between species, and specie brain region information is not 100% covered in the database(e.g. we don't have yet studies for Human Striatum, but do have for Mouse and Rat), that's why when querying the database for brain region information you will need to specify the specie. The full list of genes for specific region could be obtained wuth command getAllGenes4BrainRegion, which returns the table with main gene identifiers, like internal Gene IDs, MGI

ID, Human Entrez ID, Human Gene Name, Mouse Entrez ID, Mouse Gene Name, Rat Entrez ID, Rat Gene Name.

If you need to check which genes of your list were identified in specific region, you can use getGenes4BrainRegion command, which will select only genes associated with specific region from your list.

To obtain the PPI network for brain region you need to submit the list of Internal GeneIDs obtained with previous commands.

```
#getting the full list of brain regions
reg <- getBrainRegions()
pander(reg)
```

ID	Name	Description	InterlexID	ParentID
1	Brain	Whole brain	ILX:0101431	1
2	Forebrain	Whole forebrain	ILX:0104355	1
3	Midbrain	Midbrain	ILX:0106935	1
4	Cerebellum	Cerebellum	ILX:0101963	1
5	Telencephalon	Telencephalon	ILX:0111558	2
6	Hypothalamus	Hypothalamus	ILX:0105177	2
7	Hippocampus	Hippocampus	ILX:0105021	5
8	Striatum	Striatum	ILX:0111098	5
9	Cerebral cortex	Neocortex	ILX:0101978	5
10	Frontal lobe	Frontal lobe/frontal cortex	ILX:0104451	9
11	Occipital lobe	Occipital lobe	ILX:0107883	9
12	Temporal lobe	Temporal lobe	ILX:0111590	9
13	Parietal lobe	Parietal lobe	ILX:0108534	9
14	Prefrontal cortex	Prefrontal cortex	ILX:0109209	10
15	Motor cortex	Motor cortex	ILX:0107119	10
16	Visual cortex	Visual cortex	ILX:0112513	11
17	Medial cortex	Medial cortex	ILX:0106634	9
18	Caudal cortex	Caudal cortex	NA	9

```
#getting all genes for mouse Striatum
gns <- getAllGenes4BrainRegion(brainRegion = "Striatum",taxID = 10090)
pander(head(gns))
```

Таблица 31: Table continues below

GeneID	Localisation	MGI	HumanEntrez	MouseEntrez	HumanName
1	Postsynaptic	MGI:1277959	1742	13385	DLG4
2	Postsynaptic	MGI:88256	815	12322	CAMK2A
3	Postsynaptic	MGI:96568	9118	226180	INA
4	Postsynaptic	MGI:98388	6711	20742	SPTBN1
6	Postsynaptic	MGI:1344351	1740	23859	DLG2
7	Postsynaptic	MGI:95821	2904	14812	GRIN2B

MouseName	PMID	Paper	Year	SpeciesTaxID	BrainRegion
Dlg4	30071621	ROY_2018	2018	10090	Striatum
Camk2a	30071621	ROY_2018	2018	10090	Striatum

MouseName	PMID	Paper	Year	SpeciesTaxID	BrainRegion
Ina	30071621	ROY_2018	2018	10090	Striatum
Sptbn1	30071621	ROY_2018	2018	10090	Striatum
Dlg2	30071621	ROY_2018	2018	10090	Striatum
Grin2b	30071621	ROY_2018	2018	10090	Striatum

```
#getting full PPI network for postsynaptic compartment
ppi <- getPPIbyIDs4BrainRegion(
    gns$GeneID, brainRegion = "Striatum",
    taxID = 10090, type = "limited")
pander(head(ppi))
```

A	B
365	148
1048	148
52	365
52	1048
321	1048
321	365

12.Checking third-party list against Synaptic Proteome db

```
listG<-findGenesByEntrez(1:250) #check whic genes from 250 random EntrezIds are in the database
dim(listG)
#> [1] 124   8
head(listG)
#> # A tibble: 6 x 8
#>   GeneID MGI        HumanEntrez MouseEntrez RatEntrez HumanName MouseName RatName
#>    <int> <chr>            <int>       <int>     <int> <chr>     <chr>     <chr>
#> 1     34 MGI:87994          226       11674     24189 ALDOA     Aldoa     Aldoa
#> 2     35 MGI:2137~           87      109711     81634 ACTN1     Actn1     Actn1
#> 3     39 MGI:1019~          160       11771    308578 AP2A1     Ap2a1     Ap2a1
#> 4     54 MGI:87918          118       11518     24170 ADD1      Add1      Add1
#> 5     85 MGI:87919          119       11519     24171 ADD2      Add2      Add2
#> 6     97 MGI:1890~           81       60595     63836 ACTN4     Actn4     Actn4

getCompartments()
#> # A tibble: 4 x 3
#>      ID Name              Description
#>   <int> <chr>             <chr>
#> 1     1 Postsynaptic      Postsynaptic
#> 2     2 Presynaptic       Presynaptic
#> 3     3 Synaptosome       Synaptosome
#> 4     4 Synaptic_Vesicle Synaptic_Vesicle
presG <- getGenes4Compartment(listG$GeneID, 2) #check which genes from subset identified as synaptic are presynaptic
dim(presG)
#> [1] 67  8
head(presG)
#> # A tibble: 6 x 8
#>   GeneID MGI        HumanEntrez MouseEntrez RatEntrez HumanName MouseName RatName
```

```
#>    <int> <chr>            <int>      <int>     <int> <chr>     <chr>     <chr>
#> 1     34 MGI:87994         226      11674     24189 ALDOA     Aldoa     Aldoa
#> 2     35 MGI:2137~          87     109711     81634 ACTN1     Actn1     Actn1
#> 3     39 MGI:1019~         160      11771    308578 AP2A1     Ap2a1     Ap2a1
#> 4     54 MGI:87918         118      11518     24170 ADD1      Add1      Add1
#> 5     85 MGI:87919         119      11519     24171 ADD2      Add2      Add2
#> 6     97 MGI:1890~          81      60595     63836 ACTN4     Actn4     Actn4

getBrainRegions()
#> # A tibble: 18 x 5
#>       ID Name              Description                 InterlexID  ParentID
#>    <int> <chr>             <chr>                       <chr>          <int>
#>  1     1 Brain             Whole brain                 ILX:0101431        1
#>  2     2 Forebrain         Whole forebrain             ILX:0104355        1
#>  3     3 Midbrain          Midbrain                    ILX:0106935        1
#>  4     4 Cerebellum        Cerebellum                  ILX:0101963        1
#>  5     5 Telencephalon     Telencephalon               ILX:0111558        2
#>  6     6 Hypothalamus      Hypothalamus                ILX:0105177        2
#>  7     7 Hippocampus       Hippocampus                 ILX:0105021        5
#>  8     8 Striatum          Striatum                    ILX:0111098        5
#>  9     9 Cerebral cortex   Neocortex                   ILX:0101978        5
#> 10    10 Frontal lobe      Frontal lobe/frontal cortex ILX:0104451        9
#> 11    11 Occipital lobe    Occipital lobe              ILX:0107883        9
#> 12    12 Temporal lobe     Temporal lobe               ILX:0111590        9
#> 13    13 Parietal lobe     Parietal lobe               ILX:0108534        9
#> 14    14 Prefrontal cortex Prefrontal cortex           ILX:0109209       10
#> 15    15 Motor cortex      Motor cortex                ILX:0107119       10
#> 16    16 Visual cortex     Visual cortex               ILX:0112513       11
#> 17    17 Medial cortex     Medial cortex               ILX:0106634        9
#> 18    18 Caudal cortex     Caudal cortex               <NA>               9
listR <- getGenes4BrainRegion(listG$GeneID, brainRegion = "Cerebellum", taxID = 10090) #check which genes from subs
dim(listR)
#> [1] 186  12
head(listR)
#> # A tibble: 6 x 12
#>   GeneID Localisation MGI      HumanEntrez MouseEntrez HumanName MouseName   PMID
#>    <int> <chr>        <chr>          <int>       <int> <chr>     <chr>      <int>
#> 1     34 Postsynaptic MGI:87~          226       11674 ALDOA     Aldoa     1.81e7
#> 2     34 Postsynaptic MGI:87~          226       11674 ALDOA     Aldoa     3.01e7
#> 3     34 Synaptosome  MGI:87~          226       11674 ALDOA     Aldoa     3.20e7
#> 4     34 Synaptosome  MGI:87~          226       11674 ALDOA     Aldoa     3.21e7
#> 5     35 Postsynaptic MGI:21~           87      109711 ACTN1     Actn1     1.81e7
#> 6     35 Postsynaptic MGI:21~           87      109711 ACTN1     Actn1     3.01e7
#> # ... with 4 more variables: Paper <chr>, Year <int>, SpeciesTaxID <int>,
#> #   BrainRegion <chr>
```

13.Visualisatiion of PPI network with Igraph.

Combine information from PPI data.frame obtained with functions like getPPIbyName, getPPIbyEntrez, getPPIbyIDs4Compartment or getPPIbyIDs4BrainRegion with information about genes obtained from getGenesByID to make interpretable undirected PPI graph in igraph format. In this format network could be further analysed and visualized by algorithms from igraph package.

```
library(igraph)
#>
#> Attaching package: 'igraph'
#> The following objects are masked from 'package:dplyr':
#>
#>     as_data_frame, groups, union
#> The following objects are masked from 'package:BiocGenerics':
#>
#>     normalize, path, union
#> The following objects are masked from 'package:stats':
#>
#>     decompose, spectrum
#> The following object is masked from 'package:base':
#>
#>     union
g<-getIGraphFromPPI(
    getPPIbyIDs(c(48, 129,  975,  4422, 5715, 5835), type='lim'))
plot(g,vertex.label=V(g)$RatName,vertex.size=25)
```

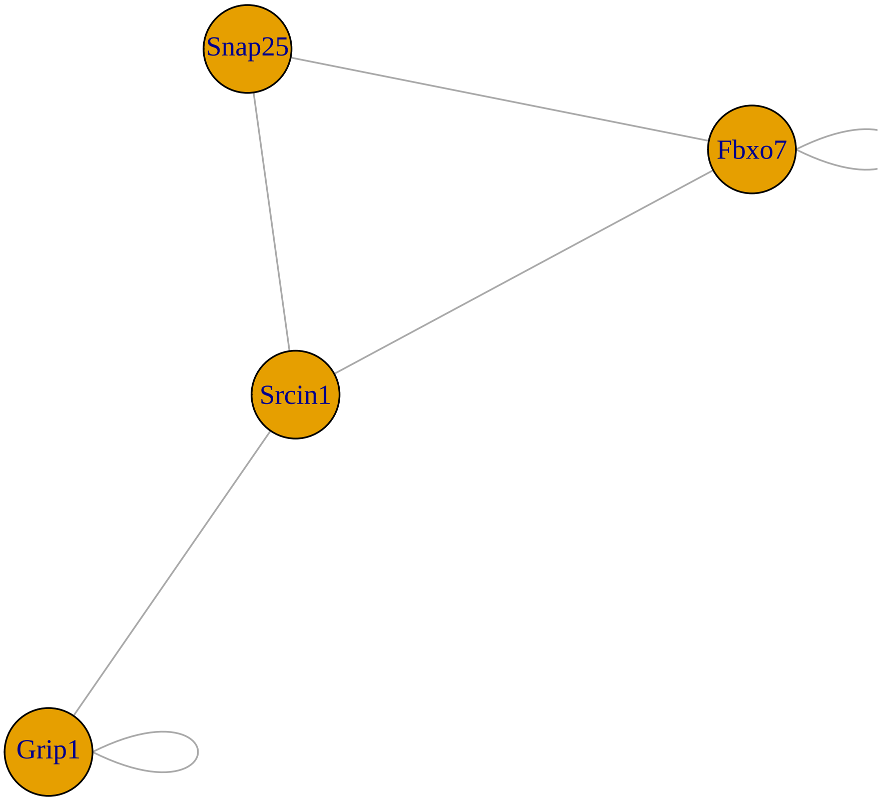

1.Export of PPI network as a table.

If Igraph is not an option, the PPI network could be exported as an interpretible table to be processed with other tools, e.g. Cytoscape,etc.

```
tbl<-getTableFromPPI(getPPIbyIDs(c(48, 585, 710), type='limited'))
tbl
#> # A tibble: 2 x 16
#>       A     B MGI.A     HumanEntrez.A MouseEntrez.A RatEntrez.A HumanName.A
```

```
#>   <int> <int> <chr>          <int>       <int>      <int> <chr>
#> 1   710   710 MGI:95602       2534        14360      25150 FYN
#> 2   585   585 MGI:98397       6714        20779      83805 SRC
#> # ... with 9 more variables: MouseName.A <chr>, RatName.A <chr>, MGI.B <chr>,
#> #   HumanEntrez.B <int>, MouseEntrez.B <int>, RatEntrez.B <int>,
#> #   HumanName.B <chr>, MouseName.B <chr>, RatName.B <chr>
```

References

Appendix

Versions

Session Info

version	R version 4.1.2 (2021-11-01)
os	macOS Catalina 10.15.7
system	x86_64, darwin17.0
ui	X11
language	(EN)
collate	en_GB.UTF-8
ctype	en_GB.UTF-8
tz	Europe/London
date	2022-10-21
pandoc	2.18 @ /Applications/RStudio.app/Contents/MacOS/quarto/bin/tools/ (via rmarkdown)

Таблица 35: Table continues below

	package	ondiskversion
AnnotationDbi	AnnotationDbi	1.56.2
AnnotationHub	AnnotationHub	3.2.2
assertthat	assertthat	0.2.1
Biobase	Biobase	2.54.0
BiocFileCache	BiocFileCache	2.2.1
BiocGenerics	BiocGenerics	0.40.0
BiocManager	BiocManager	1.30.18
BiocVersion	BiocVersion	3.14.0
Biostrings	Biostrings	2.62.0
bit	bit	4.0.4
bit64	bit64	4.0.5
bitops	bitops	1.0.7
blob	blob	1.2.3
cachem	cachem	1.0.6
callr	callr	3.7.0
cli	cli	3.3.0
colorspace	colorspace	2.0.3
crayon	crayon	1.5.1
curl	curl	4.3.2
DBI	DBI	1.1.3
dbplyr	dbplyr	2.2.1

	package	ondiskversion
devtools	devtools	2.4.3
digest	digest	0.6.29
dplyr	dplyr	1.0.9
ellipsis	ellipsis	0.3.2
evaluate	evaluate	0.15
fansi	fansi	1.0.3
fastmap	fastmap	1.1.0
filelock	filelock	1.0.2
fs	fs	1.5.2
generics	generics	0.1.2
GenomeInfoDb	GenomeInfoDb	1.30.1
GenomeInfoDbData	GenomeInfoDbData	1.2.7
ggplot2	ggplot2	3.3.6
glue	glue	1.6.2
gtable	gtable	0.3.0
htmltools	htmltools	0.5.2
httpuv	httpuv	1.6.5
httr	httr	1.4.3
igraph	igraph	1.3.2
interactiveDisplayBase	interactiveDisplayBase	1.32.0
IRanges	IRanges	2.28.0
KEGGREST	KEGGREST	1.34.0
knitr	knitr	1.39
later	later	1.3.0
lifecycle	lifecycle	1.0.1
magrittr	magrittr	2.0.3
memoise	memoise	2.0.1
mime	mime	0.12
munsell	munsell	0.5.0
pander	pander	0.6.5
pillar	pillar	1.7.0
pkgbuild	pkgbuild	1.3.1
pkgconfig	pkgconfig	2.0.3
pkgload	pkgload	1.3.0
png	png	0.1.7
prettyunits	prettyunits	1.1.1
processx	processx	3.6.1
promises	promises	1.2.0.1
ps	ps	1.7.1
purrr	purrr	0.3.4
R6	R6	2.5.1
rappdirs	rappdirs	0.3.3
rbibutils	rbibutils	2.2.8
Rcpp	Rcpp	1.0.8.3
RCurl	RCurl	1.98.1.7
Rdpack	Rdpack	2.3.1
remotes	remotes	2.4.2
rlang	rlang	1.0.3
rmarkdown	rmarkdown	2.14
RSQLite	RSQLite	2.2.14
rstudioapi	rstudioapi	0.13
S4Vectors	S4Vectors	0.32.4

	package	ondiskversion
scales	scales	1.2.0
sessioninfo	sessioninfo	1.2.2
shiny	shiny	1.7.1
stringi	stringi	1.7.6
stringr	stringr	1.4.0
synaptome.data	synaptome.data	0.99.3
synaptome.ldb	synaptome.ldb	0.99.9
tibble	tibble	3.1.7
tidyselect	tidyselect	1.1.2
usethis	usethis	2.1.6
utf8	utf8	1.2.2
vctrs	vctrs	0.4.1
withr	withr	2.5.0
xfun	xfun	0.31
xtable	xtable	1.8.4
XVector	XVector	0.34.0
yaml	yaml	2.3.5
zlibbioc	zlibbioc	1.40.0

Таблица 36: Table continues below

	loadedversion	attached	is_base	date
AnnotationDbi	1.56.2	FALSE	FALSE	2021-11-09
AnnotationHub	3.2.2	TRUE	FALSE	2022-03-01
assertthat	0.2.1	FALSE	FALSE	2019-03-21
Biobase	2.54.0	FALSE	FALSE	2021-10-26
BiocFileCache	2.2.1	TRUE	FALSE	2022-01-23
BiocGenerics	0.40.0	TRUE	FALSE	2021-10-26
BiocManager	1.30.18	FALSE	FALSE	2022-05-18
BiocVersion	3.14.0	FALSE	FALSE	2021-05-19
Biostrings	2.62.0	FALSE	FALSE	2021-10-26
bit	4.0.4	FALSE	FALSE	2020-08-04
bit64	4.0.5	FALSE	FALSE	2020-08-30
bitops	1.0-7	FALSE	FALSE	2021-04-24
blob	1.2.3	FALSE	FALSE	2022-04-10
cachem	1.0.6	FALSE	FALSE	2021-08-19
callr	3.7.0	FALSE	FALSE	2021-04-20
cli	3.3.0	FALSE	FALSE	2022-04-25
colorspace	2.0-3	FALSE	FALSE	2022-02-21
crayon	1.5.1	FALSE	FALSE	2022-03-26
curl	4.3.2	FALSE	FALSE	2021-06-23
DBI	1.1.3	FALSE	FALSE	2022-06-18
dbplyr	2.2.1	TRUE	FALSE	2022-06-27
devtools	2.4.3	FALSE	FALSE	2021-11-30
digest	0.6.29	FALSE	FALSE	2021-12-01
dplyr	1.0.9	TRUE	FALSE	2022-04-28
ellipsis	0.3.2	FALSE	FALSE	2021-04-29
evaluate	0.15	FALSE	FALSE	2022-02-18
fansi	1.0.3	FALSE	FALSE	2022-03-24
fastmap	1.1.0	FALSE	FALSE	2021-01-25
filelock	1.0.2	FALSE	FALSE	2018-10-05

	loadedversion	attached	is_base	date
fs	1.5.2	FALSE	FALSE	2021-12-08
generics	0.1.2	FALSE	FALSE	2022-01-31
GenomeInfoDb	1.30.1	FALSE	FALSE	2022-01-30
GenomeInfoDbData	1.2.7	FALSE	FALSE	2022-03-02
ggplot2	3.3.6	TRUE	FALSE	2022-05-03
glue	1.6.2	FALSE	FALSE	2022-02-24
gtable	0.3.0	FALSE	FALSE	2019-03-25
htmltools	0.5.2	FALSE	FALSE	2021-08-25
httpuv	1.6.5	FALSE	FALSE	2022-01-05
httr	1.4.3	FALSE	FALSE	2022-05-04
igraph	1.3.2	TRUE	FALSE	2022-06-13
interactiveDisplayBase	1.32.0	FALSE	FALSE	2021-10-26
IRanges	2.28.0	FALSE	FALSE	2021-10-26
KEGGREST	1.34.0	FALSE	FALSE	2021-10-26
knitr	1.39	TRUE	FALSE	2022-04-26
later	1.3.0	FALSE	FALSE	2021-08-18
lifecycle	1.0.1	FALSE	FALSE	2021-09-24
magrittr	2.0.3	FALSE	FALSE	2022-03-30
memoise	2.0.1	FALSE	FALSE	2021-11-26
mime	0.12	FALSE	FALSE	2021-09-28
munsell	0.5.0	FALSE	FALSE	2018-06-12
pander	0.6.5	TRUE	FALSE	2022-03-18
pillar	1.7.0	FALSE	FALSE	2022-02-01
pkgbuild	1.3.1	FALSE	FALSE	2021-12-20
pkgconfig	2.0.3	FALSE	FALSE	2019-09-22
pkgload	1.3.0	FALSE	FALSE	2022-06-27
png	0.1-7	FALSE	FALSE	2013-12-03
prettyunits	1.1.1	FALSE	FALSE	2020-01-24
processx	3.6.1	FALSE	FALSE	2022-06-17
promises	1.2.0.1	FALSE	FALSE	2021-02-11
ps	1.7.1	FALSE	FALSE	2022-06-18
purrr	0.3.4	FALSE	FALSE	2020-04-17
R6	2.5.1	FALSE	FALSE	2021-08-19
rappdirs	0.3.3	FALSE	FALSE	2021-01-31
rbibutils	2.2.8	FALSE	FALSE	2022-04-11
Rcpp	1.0.8.3	FALSE	FALSE	2022-03-17
RCurl	1.98-1.7	FALSE	FALSE	2022-06-09
Rdpack	2.3.1	FALSE	FALSE	2022-06-07
remotes	2.4.2	FALSE	FALSE	2021-11-30
rlang	1.0.3	FALSE	FALSE	2022-06-27
rmarkdown	2.14	FALSE	FALSE	2022-04-25
RSQLite	2.2.14	FALSE	FALSE	2022-05-07
rstudioapi	0.13	FALSE	FALSE	2020-11-12
S4Vectors	0.32.4	FALSE	FALSE	2022-03-29
scales	1.2.0	FALSE	FALSE	2022-04-13
sessioninfo	1.2.2	FALSE	FALSE	2021-12-06
shiny	1.7.1	FALSE	FALSE	2021-10-02
stringi	1.7.6	FALSE	FALSE	2021-11-29
stringr	1.4.0	FALSE	FALSE	2019-02-10
synaptome.data	0.99.3	TRUE	FALSE	2022-03-02
synaptome.ldb	0.99.9	TRUE	FALSE	2022-10-13
tibble	3.1.7	FALSE	FALSE	2022-05-03

	loadedversion	attached	is_base	date
tidyselect	1.1.2	FALSE	FALSE	2022-02-21
usethis	2.1.6	FALSE	FALSE	2022-05-25
utf8	1.2.2	FALSE	FALSE	2021-07-24
vctrs	0.4.1	FALSE	FALSE	2022-04-13
withr	2.5.0	FALSE	FALSE	2022-03-03
xfun	0.31	FALSE	FALSE	2022-05-10
xtable	1.8-4	FALSE	FALSE	2019-04-21
XVector	0.34.0	FALSE	FALSE	2021-10-26
yaml	2.3.5	FALSE	FALSE	2022-02-21
zlibbioc	1.40.0	FALSE	FALSE	2021-10-26

	source
AnnotationDbi	Bioconductor
AnnotationHub	Bioconductor
assertthat	CRAN (R 4.1.0)
Biobase	Bioconductor
BiocFileCache	Bioconductor
BiocGenerics	Bioconductor
BiocManager	CRAN (R 4.1.2)
BiocVersion	Bioconductor
Biostrings	Bioconductor
bit	CRAN (R 4.1.0)
bit64	CRAN (R 4.1.0)
bitops	CRAN (R 4.1.0)
blob	CRAN (R 4.1.2)
cachem	CRAN (R 4.1.0)
callr	CRAN (R 4.1.0)
cli	CRAN (R 4.1.2)
colorspace	CRAN (R 4.1.2)
crayon	CRAN (R 4.1.2)
curl	CRAN (R 4.1.0)
DBI	CRAN (R 4.1.2)
dbplyr	CRAN (R 4.1.2)
devtools	CRAN (R 4.1.0)
digest	CRAN (R 4.1.0)
dplyr	CRAN (R 4.1.2)
ellipsis	CRAN (R 4.1.0)
evaluate	CRAN (R 4.1.2)
fansi	CRAN (R 4.1.2)
fastmap	CRAN (R 4.1.0)
filelock	CRAN (R 4.1.0)
fs	CRAN (R 4.1.0)
generics	CRAN (R 4.1.2)
GenomeInfoDb	Bioconductor
GenomeInfoDbData	Bioconductor
ggplot2	CRAN (R 4.1.2)
glue	CRAN (R 4.1.2)
gtable	CRAN (R 4.1.0)
htmltools	CRAN (R 4.1.0)
httpuv	CRAN (R 4.1.2)
httr	CRAN (R 4.1.2)

	source
igraph	CRAN (R 4.1.2)
interactiveDisplayBase	Bioconductor
IRanges	Bioconductor
KEGGREST	Bioconductor
knitr	CRAN (R 4.1.2)
later	CRAN (R 4.1.0)
lifecycle	CRAN (R 4.1.0)
magrittr	CRAN (R 4.1.2)
memoise	CRAN (R 4.1.0)
mime	CRAN (R 4.1.0)
munsell	CRAN (R 4.1.0)
pander	CRAN (R 4.1.2)
pillar	CRAN (R 4.1.2)
pkgbuild	CRAN (R 4.1.0)
pkgconfig	CRAN (R 4.1.0)
pkgload	CRAN (R 4.1.2)
png	CRAN (R 4.1.0)
prettyunits	CRAN (R 4.1.0)
processx	CRAN (R 4.1.2)
promises	CRAN (R 4.1.0)
ps	CRAN (R 4.1.2)
purrr	CRAN (R 4.1.0)
R6	CRAN (R 4.1.0)
rappdirs	CRAN (R 4.1.0)
rbibutils	CRAN (R 4.1.2)
Rcpp	CRAN (R 4.1.2)
RCurl	CRAN (R 4.1.2)
Rdpack	CRAN (R 4.1.2)
remotes	CRAN (R 4.1.0)
rlang	CRAN (R 4.1.2)
rmarkdown	CRAN (R 4.1.2)
RSQLite	CRAN (R 4.1.2)
rstudioapi	CRAN (R 4.1.0)
S4Vectors	Bioconductor
scales	CRAN (R 4.1.2)
sessioninfo	CRAN (R 4.1.0)
shiny	CRAN (R 4.1.0)
stringi	CRAN (R 4.1.0)
stringr	CRAN (R 4.1.0)
synaptome.data	Bioconductor
synaptome.ldb	Bioconductor
tibble	CRAN (R 4.1.2)
tidyselect	CRAN (R 4.1.2)
usethis	CRAN (R 4.1.2)
utf8	CRAN (R 4.1.0)
vctrs	CRAN (R 4.1.2)
withr	CRAN (R 4.1.2)
xfun	CRAN (R 4.1.2)
xtable	CRAN (R 4.1.0)
XVector	Bioconductor
yaml	CRAN (R 4.1.2)
zlibbioc	Bioconductor
