# Supplementary figures and images for: Synaptome.db: a bioconductor package for synaptic proteomics data
Source: Bioinform Adv. 2022 Nov 12;2(1):vbac086. doi: 10.1093/bioadv/vbac086 (PMC9710567; doi:10.1093/bioadv/vbac086)

# Alzheimer's\_disease\_genes

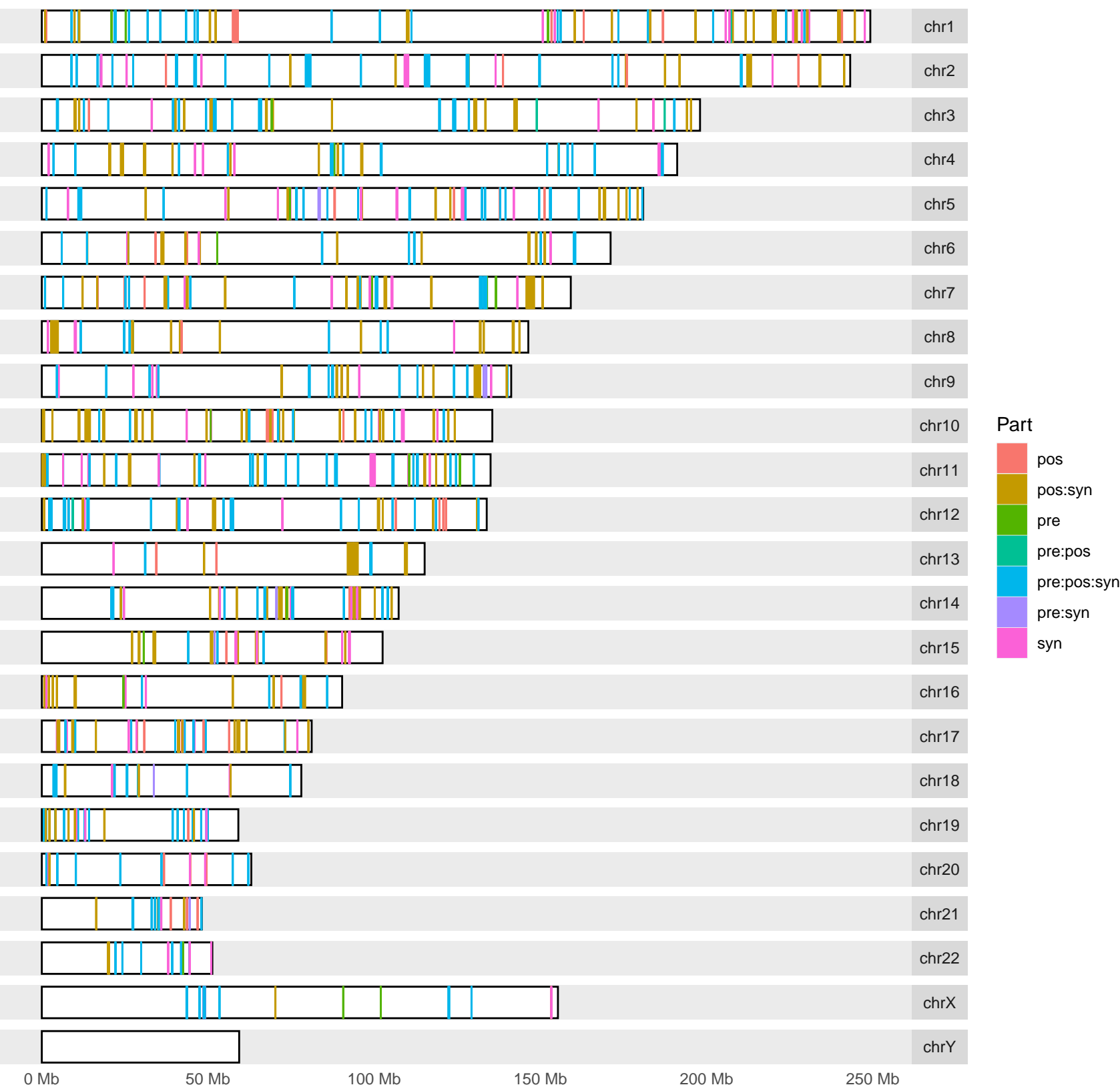

Supplement: vbac086_supplementary_data [file vbac086_supplementary_data.zip › suppl_data/Supplementary Fiigure 1.pdf]
